# A precise language network revealed by the independent component-based lesion mapping in post-stroke aphasia

**DOI:** 10.3389/fneur.2022.981653

**Published:** 2022-09-30

**Authors:** Weijing Ren, Chunying Jia, Ying Zhou, Jingdu Zhao, Bo Wang, Weiyong Yu, Shiyi Li, Yiru Hu, Hao Zhang

**Affiliations:** ^1^School of Rehabilitation, Capital Medical University, Beijing, China; ^2^Department of Neurorehabilitation, China Rehabilitation Research Center, Beijing Bo'ai Hospital, Beijing, China; ^3^University of Health and Rehabilitation Sciences, Qingdao, China; ^4^Beijing Institute for Brain Disorders, Capital Medical University, Beijing, China; ^5^Department of Hearing and Language Rehabilitation, China Rehabilitation Research Center, Beijing Bo'ai Hospital, Beijing, China; ^6^Department of Radiology, China Rehabilitation Research Center, Beijing Bo'ai Hospital, Beijing, China

**Keywords:** functional magnetic resonance imaging, lesion network mapping, independent component analysis, aphasia, stroke, language network

## Abstract

Brain lesion mapping studies have provided the strongest evidence regarding the neural basis of cognition. However, it remained a problem to identify symptom-specific brain networks accounting for observed clinical and neuroanatomical heterogeneity. Independent component analysis (ICA) is a statistical method that decomposes mixed signals into multiple independent components. We aimed to solve this issue by proposing an independent component-based lesion mapping (ICLM) method to identify the language network in patients with moderate to severe post-stroke aphasia. Lesions were first extracted from 49 patients with post-stroke aphasia as masks applied to fMRI data in a cohort of healthy participants to calculate the functional connectivity (FC) within the masks and non-mask brain voxels. ICA was further performed on a reformatted FC matrix to extract multiple independent networks. Specifically, we found that one of the lesion-related independent components (ICs) highly resembled classical language networks. Moreover, the damaged level within the language-related lesioned network is strongly associated with language deficits, including aphasia quotient, naming, and auditory comprehension scores. In comparison, none of the other two traditional lesion mapping methods found any regions responsible for language dysfunction. The language-related lesioned network extracted with the ICLM method showed high specificity in detecting aphasia symptoms compared with the performance of resting ICs and classical language networks. In total, we detected a precise language network in patients with aphasia and proved its efficiency in the relationship with language symptoms. In general, our ICLM could successfully identify multiple lesion-related networks from complicated brain diseases, and be used as an effective tool to study brain-behavior relationships and provide potential biomarkers of particular clinical behavioral deficits.

## Introduction

Mapping the neural circuits of a specific neurological or psychiatric symptom is crucial for neuroscience research and clinical practice (1, 2). From the earliest history of cognitive neuroscience to modern neurology, detailed studies on focal brain lesions have provided the strongest evidence to localize the neuroanatomical substrate of cognition (3–7). However, because of the challenging clinical and neuroanatomical heterogeneities in many brain diseases, it remains highly infeasible to dissociate the precise brain circuit responsible for a specific neurologic symptom. Mapping the causal links between symptoms and neuroanatomy for patients with complex neurologic and psychiatric disorders remains an unsolved problem (8, 9).

Contemporary neuroimaging techniques have been applied to patients with cognitive deficits following brain damage to examine the functional and anatomical organizations of the lesioned brain (10, 11). The traditional voxel-based lesion-symptom mapping (VLSM) method based on the assumption of “collective lesioned voxels” is aimed to find the most commonly damaged voxels associated with symptom severity (12). Despite the seemingly high spatial resolution, VLSM has been recognized as apparently flawed with the rise of network theories and evidence of many symptoms caused by damage distributed in different brain locations (13, 14).

Lesion network mapping (LNM) was then proposed linking brain lesions to a shared network of connected regions (15, 16). Specifically, LNM took each patient's lesion mask as a “seed” to generate the likely lesion-affected functional connectome based on a cohort of healthy controls. The symptom-related network was identified as the shared functional connectome by overlaying connectome-based maps across patients (15). This method has been successfully applied to various symptoms such as hallucinations, delusions, abnormal movements, and loss of consciousness, with obvious advantages in revealing the brain circuit of a single symptom with large heterogeneity in lesion locations (6, 17–23). However, the plain overlaying approach to construct the functional network was also likely to cause low specificity (24). When connectivity patterns are broadly diverse across patients, as in those with multiple lesion-induced symptoms, it is not clear whether meaningful information could be obtained from the shared connectome. As patients seldom converge into only one deficit, the affected brain regions would likely belong to multiple functions instead of a single one, and the subsequent overlaying might yield a mixed non-sense network that failed to explain any particular symptom.

To illustrate, stroke, a major cause of severe disability in adults, broadly influences multifarious cognitive functions (25–27). As one of the most common clinical features affecting about one-third of patients with stroke, aphasia often co-occurs with other cognitive behavioral problems such as motion, attention, or memory deficits (28). Due to the comorbidity with different lesioned cognitive networks, previous studies utilizing the LNM method were questioned about the specificity of the obtained network in the causal relationship with the aphasia symptom (29, 30). Hence, a more precise network segregation technique is desperately required to embrace the complexity of lesion topographies as well as clinical symptoms. Ensuring both the sensitivity and specificity of the lesioned-mapping method is also an essential requirement to comprehensively study the network-behavior relationships in complicated brain diseases and to find the accurate brain biomarkers of a specific cognitive deficit.

A feasible way is to apply the independent component analysis (ICA), a generative machine learning method commonly used for blind source separation in fields of neural networks and signal processing (31). The ICA method has been employed in brain imaging data to extract multiple spatially independent networks from mixed BOLD signals (32). With an emphasis on blind decomposition, the ICA method is conventionally performed on healthy brains, detecting some general networks such as visual, sensorimotor, and default mode networks (8, 33–35). As a drawback, the ICA method often fails to find a more subtle functional network with a high correlation with a specific task (36). One critical account might be that whole brain signals involve too much mixed and irrelevant information to separate a fine-grained function without any prior spatial constraints. Therefore, a more accurate way should be to take advantage of both the ICA and lesioned brains. With the lesion-affected connectome being selectively identified, the scope of decomposing source signals can be narrowed more specifically. With better segregation of a particular cognitive network, we would be more efficient in establishing linkages with related symptoms and in delineating lesion-associated functional networks.

Here, we propose a method by leveraging independent component-based lesion mapping (ICLM) to precisely identify a functional network that is specifically related to a symptom in patients with brain injury. We chose patients with post-stroke aphasia as the target population mainly for two reasons. First, large variability of lesion site and lesion complexity is commonly observed in this population (37); thus, we could compare whether and how our method could outperform the two established lesion mapping methods (VLSM and LNM) in diseases with high heterogeneity. Moreover, the language network is a well-studied function that is suitable for verifying our identified functional map (33, 38). Specifically, we first demonstrated how the heterogeneity in lesion locations could affect the results from the two established LM methods. Next, we present our method by identifying the language network by ICLM analysis. Then, the damage level of the language network was correlated with aphasia symptoms to test the specificity of our method compared with other networks. Finally, we performed ICLM independently on randomly divided subgroups of participants to test the reproducibility of the obtained language network.

## Materials and methods

### Participants

A total of 49 patients hospitalized in the Department of Neurorehabilitation of the China Rehabilitation Research Center were recruited for this study. The study was according to the Declaration of Helsinki ethics principles and approved by the Ethics Committee of the China Rehabilitation Research Center. All the patients gave written informed consent before participation.

The patients were recruited following these inclusion criteria: (1) had a stroke due to a single infarction or hemorrhage in the left hemisphere, (2) were diagnosed with aphasia according to the Chinese version of the Western Aphasia Battery (WAB) and had an aphasia quotient of < 93.8 (39), (3) using Chinese as mother tongue, (4) were right-handed. The exclusion criteria were (1) having visual and hearing impairments that affect language evaluation, (2) with metallic foreign bodies or other implanted electronic devices so could not undergo MRI examinations, (3) having language disorder or severe dysarthria before the stroke, (4) comorbidity with other neurological diseases that are associated with speech disorders.

The healthy control cohort was selected from the Beijing Normal University of the Consortium for Reliability and Reproducibility (CoRR-BNU) dataset with 50 participants (23 males, mean age = 23.39, range 19–30) (http://fcon_1000.projects.nitrc.org). All the participants had no previous history of nervous system or mental diseases and had signed a written informed consent form before getting scanned. The study was approved by the Institutional Review Board of Beijing Normal University Imaging Center for Brain Research.

### Language assessments for patients

The assessments were evaluated by a professional speech-language pathologist after patients' enrollment using the Chinese version of the WAB. The WAB assessment for this study included four subsets: naming, auditory comprehension, fluency, and repetition. The Aphasia Quotient (AQ) (range 0–100) is a composite score of the WAB and was calculated using the formula developed by Kertesz (naming score/10 + comprehension score/20 + fluency score + repetition score/10) × 2 (40–42), with lower scores indicating a more severe deficit in language. All the language assessments were performed within 3 days of MRI scanning. No new cerebral hemorrhage or infarction occurred in any patient within these days.

### Image acquisition and preprocessing

Structural images of the patients were acquired with Philips Ingenia 3.0T Magnetic Resonance System at the China Rehabilitation Research Center. Acquisition parameters were as follows: T1-weighted: TR = 7.1 ms, TE = 3.2 ms, flip angle (FA) = 7°, 192 slices with a slice thickness of 1 mm and a 0-mm slice gap, field of view (FOV) = 256 mm, and matrix size = 256 × 256. T2-weighted: TR = 2,500 ms, TE = 252 ms, FA = 90°, 192 slices with a slice thickness of 1 mm and a 0-mm slice gap, FOV = 256 mm, and matrix size = 256 × 256.

The fMRI data of healthy controls in the CoRR-BNU dataset were acquired with a SIEMENS TRIO 3-Tesla scanner at the Beijing Normal University Imaging Center for Brain Research. Each participant received four resting-state fMRI scanning sessions, with each session lasting for 8 min. During the resting-state sessions, the participants were required to keep still and not think systematically. The acquisition parameters were as follows: TR = 2,000 ms, TE = 30 ms, FA = 90°, FOV = 200 mm × 200 mm, number of slices = 33, thickness/gap = 3/0.6 mm, and in-plane resolution = 64 × 64.

The resting-state fMRI data of the 50 healthy participants were preprocessed using procedures previously described (43–47). The following steps were performed: (1) slice timing correction (SPM2; Welcome Department of Cognitive Neurology, London, United Kingdom), (2) rigid body correction for head motion with the FSL package, (3) normalization for global mean signal intensity across sessions, and (4) band-pass temporal filtering (0.01–0.08 Hz), head motion regression, whole-brain signal regression, and ventricular and white matter signal regression.

### Lesion mapping and lesion variability analysis

For each structural magnetic resonance imaging of the patients, an experienced neurologist identified and manually drew lesioned areas using MRIcron, an open-source tool for brain imaging visualization and definition of volumes of interest (http://people.cas.sc.edu/rorden/mricron/index.html) (48). Two professional neurologists reviewed the lesion segmentation. Lesion masks were spatially normalized to the Montreal Neurological Institute (MNI) 152 atlas space (voxel size = 4 mm, dimension = 64 × 64 × 64, number of voxels = 262,144) and binarized such that voxels inside the lesion had a value of 1 and all other voxels had a value of 0.

For lesion variability analysis, we combined all the 49 lesion masks to create an overlapped lesion map with a coarse view of lesion locations. Then, we calculated the proportion of commonly shared lesion regions according to different numbers of patients. The relationship between the proportion of typical lesioned patients and overlapped regions was delineated to quantify the inter-subject variability in aphasia lesion areas.

### Traditional VLSM and LNM analysis

For the standard VLSM method (12), first, we identified the voxels with at least 25% and at most 75% of all 49 patients that are lesioned, which are 1,176 in total. Then, for each voxel, we classified the participants into two groups based on the criterion below: the participants are lesioned or not lesioned in this voxel. Then, we performed an independent two-sample *t*-test on the two groups' AQ scores to build a relationship between the voxel and the symptom. We then repeated the *t*-test for all the 1,176 voxels and performed a False Discovery Rate (FDR) correction on 1,176 *p*-values. Corrected *p* < 0.05 were defined as significant, and the corresponding *t*-values were projected to the brain. In doing so, we got a *t*-map that linked the lesion and the aphasia-related symptom.

For the standard LNM method (15), the lesion masks extracted from the 49 patients were separately used to calculate the functional connectivity (FC) associated with the lesion area using the fMRI data from the 50 healthy controls. First, we used the intersection of brain areas extracted from the 50 HCs to be the brain mask, with a size of 1 × 32,894, which will be used later. Then, for the lesion mask from an individual patient, we used it as a seed ROI to calculate the Pearson correlations between the averaged time course within the ROI with all the brain voxels outside the ROI, which resulted in a lesion-affected FC matrix with a size of 50 × *VL* (*VL* = 32,894 minus number of lesioned voxels) for each patient. Correlation values were transformed into *Z*-values by Fisher-*Z* transformation. Note that each patient's size in the second dimension of the FC matrix might differ because of the variance in lesion sizes. Next, for each patient's FC matrix, we conducted a one-sample *t*-test across the 50 HCs to get the *t*-value and *p*-value for each voxel in the matrix. We then conducted a voxel-level family-wise error (FWE) correction for all the *VL p*-values, and corrected *p* < 10^−6^ were considered significant voxels (49). The corresponding *t*-values were then projected to the brain, forming a *t*-map for each patient representing the individual lesion network map. Lastly, we binarized the partial *t*-map that only considered positive values for each patient and overlaid all positive *t*-maps to identify areas that were functionally connected to the lesion sites. We used different thresholds to retain only voxels identified in most patients (i.e., 70, 80, and 90%) to get a common map (49).

### Independent component-based lesion mapping

The ICLM method was consistent with the standard LNM method in calculating the lesion-affected FC matrix for each lesion mask. The preprocessed fMRI data of the 50 healthy participants were used for calculating the FC with the lesion mask extracted from each of the 49 patients.

In doing so, we had 50 FC vectors for each patient. To preserve more spatial information in the following ICA, we then averaged the FC vectors across the 50 HCs instead of binarizing the statistical *t*-map in the traditional LNM method. The averaged FC vector represented the FC pattern with each patient's lesion site mapped in the normative fMRI data. We then combined all the 49 FC vectors to get a union FC matrix as an input dataset for ICA, with empty elements filled with zeroes. The values in the FC matrix were *Z*-scored to have a zero mean and unit variance before the ICA.

ICA seeks to recover latent sources with the assumption that observations are linearly mixed by independent latent sources (50). Given a dataset X ∈ℝ^M × V^, which is comprised of M participants and V samples (e.g., voxels), the generative model for noiseless ICA can be written as:


(1) $$\begin{array}{l} \text{X}=\text{AS}, \end{array}$$

where A ∈ℝ^M × M^ is a full rank square mixing matrix and S ∈ℝ^M × V^ is the latent source matrix. The goal of ICA is to estimate the demixing matrix W ∈ℝ^M × M^, such that the estimated source matrix Ŝ can be computed as:


(2) $$\begin{array}{l} Ŝ=WX, \end{array}$$

Because of the effects of additive noise, it is desirable to reduce the observed data to a lower-dimensional signal subspace before performing an ICA. We conducted a principal component analysis (PCA) to extract the signal subspace in this study. The knee point method was used on the cumulative explained variance to determine the order of the subspace. We then performed an ICA on the signal subspace to get independent components (ICs). The MATLAB code provided in the group ICA of fMRI Toolbox (https://trendscenter.org/software/gift/) was used to implement the ICA.

In this study, we used Infomax, an ICA algorithm widely used in biomedical imaging (51). Since ICA is an iterative algorithm, its optimization yields different solutions depending on initialization. Therefore, we performed an ICA for 30 independent runs with different random initializations and selected the most consistent run using a metric called cross inter-symbol interference (cross-ISI) (52).

The resulting ICs were then converted to *Z*-scores with a zero mean and unit variance. The *Z*-maps were thresholded at a *Z*-value of ±1 (i.e., |*Z*| > 1) and referred to as lesion-related network maps (53). These spatial maps should contain a “lesioned language network” that linked lesions and aphasia symptoms we are mainly interested in, as well as other networks related to dysfunctions in cognitive domains apart from language.

### Identification of the language network

To identify the language network from the seven ICs obtained in the ICLM, we compared the pattern of each IC with two classical language networks. One was the healthy language network defined in Yeo 17 networks parcellated from the resting-state imaging of healthy participants (44). Another was the large-sample refined language network derived from the meta-analysis results of 1,101 studies, including task-related imaging and patient data (www.neurosynth.org). We regarded the former as a healthy analog and the latter as the “gold standard.” Specifically, we calculated the dice coefficient (54) and the Pearson correlation coefficient between each positive IC map and the language region from the Yeo 17 parcellation or the language region from the Neurosynth study. The dice coefficient was used to assess the degree of spatial overlap between different maps (54). The Pearson correlation coefficient was a reflection of similarity in activation patterns between different maps. The IC that showed the highest dice coefficients and correlation coefficients was defined as the specific lesioned language network in patients with aphasia.

### Calculation of network damage score and correlation with aphasia symptoms

In order to confirm the link of language network extracted above by ICLM and aphasia-related symptoms, we examined the relationship between the AQs and the level of damage in the lesioned network across patients. Specifically, we assigned each patient a “network damage score” using the lesioned language network. To measure an individual patient's network damage score, we first identified the intersection of the binarized lesioned language map and the patient's lesion mask. Then, we extracted the maximum *Z*-value within the intersection area identified above. This *Z*-value represents the highest intensity within the area and measures the level of network damage and therefore is defined as “network damage score.”

We then explored the relationship between the network damage score and behavioral abilities. Pearson correlation coefficients were calculated between the network damage score and the behavioral scores measured by AQ. To avoid an influence caused by lesion size, we regressed out the lesion size from the network damage score before the calculation of the correlation. In addition, we also calculated the correlation coefficients between the language network damage score and the subtests scores in the WAB, i.e., naming, auditory comprehension, fluency, and repetition, to explore the relationship between the language network and performance in different language domains.

### Validation of the reproducibility, stability, and specificity of the method

To validate the robustness of our findings from the ICLM method, we mainly conducted three types of control analysis: (1) reproducibility analysis on different subgroups of patients, (2) stability analysis on different parameters in the ICLM method, and (3) specificity analysis for the network-symptom relationship.

First, to examine the reproducibility of ICLM in different subgroups, we randomly excluded one lesion and then split the remaining 48 aphasia-causing lesions into two subgroups (group A, *N* = 24; group B, *N* = 24). Then, we performed ICLM separately for each subgroup and compared the identified language network map using a dice coefficient. We calculated pairwise dice coefficients between the acquired language networks extracted from the subgroups. The random splitting was performed 10 times, and the dice results for each time were recorded.

We also considered the influence of different clinical subtypes of patients on examining the aphasia-related lesioned network. As our cohort includes patients with different lesion types (ischemic and hemorrhagic stokes), different lesion stages from onset time, and different severity in aphasia symptoms, we repeated the ICLM on each subgroup separately to test the potential influence brought by these factors. Specifically, we grouped the patients with stroke into ischemic (*N* = 19), hemorrhagic (*N* = 30), acute/subacute (< 1 month since stroke onset, *N* = 29), chronic (> 3 months since stroke onset, *N* = 20), minor aphasia (the highest 25% with AQ > 54, *N* = 12), and severe aphasia (the lowest 25% with AQ < 20.3, *N* = 12). ICLM was conducted on each subgroup, and the language network map was identified using the same approach introduced in section Identification of the language network. To test the robustness of our method, we calculated the dice coefficient between the map obtained from each subgroup and the one we got obtained all the 49 patients.

Second, to validate the robustness of our ICLM method regarding the parameter space, we also considered two possible confounds that might influence the performance of ICA and accuracy of the extracted network. One was the sample size of the healthy cohorts to construct the FC matrices and the other was the specific threshold value in constructing the lesioned language network. Specifically, we tested if our method is robust by repeating the ICLM by including more HCs. Specifically, we formed two more extensive datasets: one dataset had 17 more HCs added from the BNU dataset, which has 67 HCs in total; the second dataset had 65 HCs added from the IPCAS dataset, which has 132 HCs in total. We then performed ICLM using the two HC datasets separately and calculated the dice coefficients between the identified language network and the one identified using 50 HCs. Moreover, we also changed the threshold to either looser (*Z* = 0.5) or stricter (*Z* = 1.5) in the construction of lesioned language network, and repeated the analysis of exploring the relationship between the corresponding lesioned language network and the behavior dysfunction.

Lastly, to validate the specificity of the symptom-network relationship established by the language lesioned network, we performed a control analysis in the same way described in 2.8 but on different maps as follows: (1) the lesioned network extracted from traditional LNM, (2) other ICs extracted by ICLM, and (3) classic language networks including the healthy language network and the large-sample refined network. Of note, as the healthy language network from the Yeo 17 networks was already binarized, we counted the number of the voxels within the intersection instead and used the *Z*-scored value as the network damage score. We then calculated the correlation of the network damage score from the lesioned network with the AQ scores and compared the correlation value of the language lesioned network and those of the control networks.

## Results

### High heterogeneity in aphasia lesion areas resulted in the failure of traditional methods

**Figure 1:**
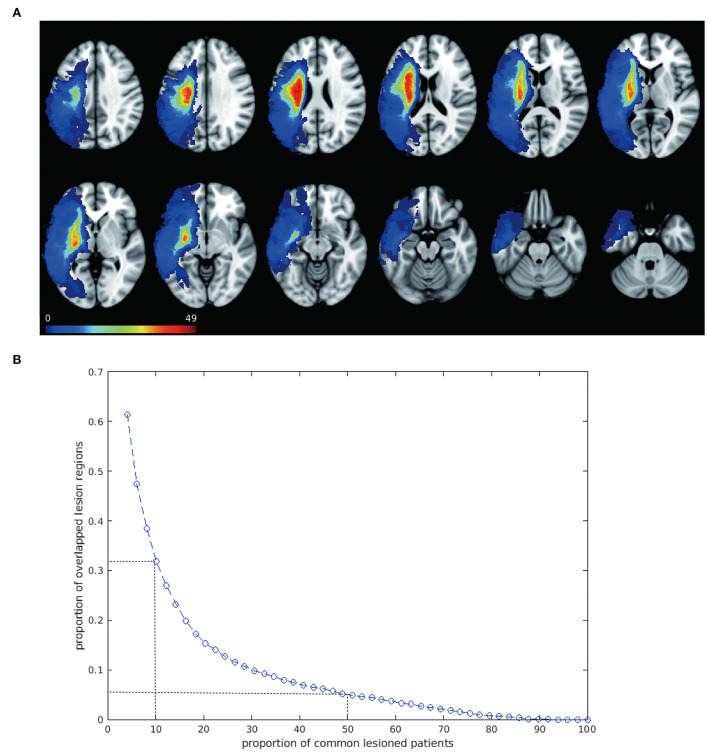
Overlapped lesion mapping shows large spatial variability. **(A)** Overlapped lesion mapping. Overlay of lesions of 49 patients with post-stroke aphasia is shown. Lesions spread to a large area of the left hemisphere and are mostly distributed in the blood supply area of the left hemisphere middle cerebral artery. Color scale illustrates the number of patients with a lesion in that voxel, with warmer color indicating higher number. **(B)** Overlap of the lesion regions across people. The blue curve shows a trend of decreasing proportion of overlapped lesion regions along with increase in patients, indicating great inter-subject variability of the lesion location. Two dots with dash lines are shown as examples: about 30% of the lesioned regions are shared by approximately 5 patients (10%); when the proportion of patients was raised to 50%, only 5% of the lesioned regions were commonly damaged.

The baseline demographic and clinical characteristics of the enrolled patients, such as gender, years of education, disease duration, type of stroke, aphasia quotient, and scores on various WAB subtests are shown in Table 1. The lesions of 49 patients with post-stroke aphasia included in this study were mostly distributed in the blood supply area of the left hemisphere middle cerebral artery, and the lesions were large and heterogeneous. All the 49 lesion masks were added together to create the overlapped lesion maps. The overlapping map of the lesions is shown in Figure 1A and exhibits a high heterogeneity in lesion locations across patients. Specifically, we found a striking decrease in the overlapped regions along with the increased number of patients (Figure 1B). While about 70% of the lesioned regions were shared in less than five patients, only 5% of the lesioned regions were collectively damaged across half of the patients. None of the lesion regions were found to be common in all the 49 patients. The analysis of the overlap between any two chosen patients also revealed about a 2/3 discrepancy (overlap proportion: mean = 0.33, std = 0.1) in their lesioned regions, indicating large inter-subject variability in the stroke lesion locations.

**Table 1:** Demographic and clinical characteristics of the patients.

**Demographics (*n* = 49)**	
Age, year	54 ± 14
Gender, female (%)	30.61%
Education, year	15 ± 4
Time since stroke onset, month	2 ± 2.88
Cortex accumulation (%)	42.86%
Hemorrhagic stroke (%)	61.22%
WAB	
Fluency	7 ± 7.5
Comprehension	115.80 ± 44.81
Repetition	45.00 ± 55.50
Naming	20.00 ± 38.50
AQ	40.10 ± 34.35
Lesion volume voxels	50,776 ± 33,697

WAB, Western Aphasia Battery; AQ, Aphasia Quotient.

Given the heterogeneity in the lesion locations, we next tested whether the traditional methods (i.e., VLSM and LNM) were able to detect a brain circuit specific to language dysfunction. Unsurprisingly, the standard VLSM failed to identify any voxels significantly associated with aphasia symptoms after multiple corrections (Supplementary Figure 1A), whereas the overlapped map in LNM decreased according to the percentage of shared participants and disappeared in the requirement of 90% shared participants, which was considered as the traditionally threshold in previous researches (Supplementary Figure 1B). Even when we loosened the threshold to calculate the overlapped map in 70 or 80% the participants, the lesioned map comprised few language-related regions and failed to detect any relationship with symptoms (*p* = 0.36).

### A specific language network identified from ICLM

**Figure 2:**
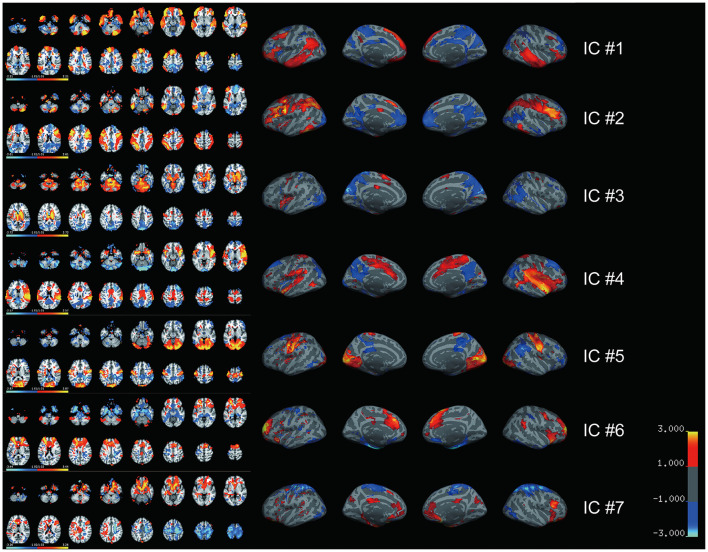
Seven independent spatial maps are generated by lesion-based independent component analysis. Seven spatially independent components derived from ICLM are illustrated with both volume (left) and surface (right) maps. The major brain regions included in each spatial map are listed. IC#1: inferior frontal gyrus, superior frontal gyrus, and middle frontal gyrus together with some other regions in premotor cortex; IC#2: fusiform gyrus, inferior parietal lobe, middle frontal gyrus, inferior frontal gyrus, and superior parietal lobule; IC#3: thalamus, pons, caudate, and precuneus; IC#4: temporal pole, insula, and supramarginal gyrus; IC#5: occipital lobe, precentral gyrus, and postcentral gyrus; IC#6: anterior cingulate, superior frontal gyrus, medial frontal gyrus, and inferior frontal gyrus; IC#7: middle frontal gyrus, orbitofrontal cortex, postcentral gyrus, precentral gyrus, and inferior frontal gyrus. Color bar indicates the weight of each voxel in the corresponding IC. IC, independent component.

**Figure 3:**
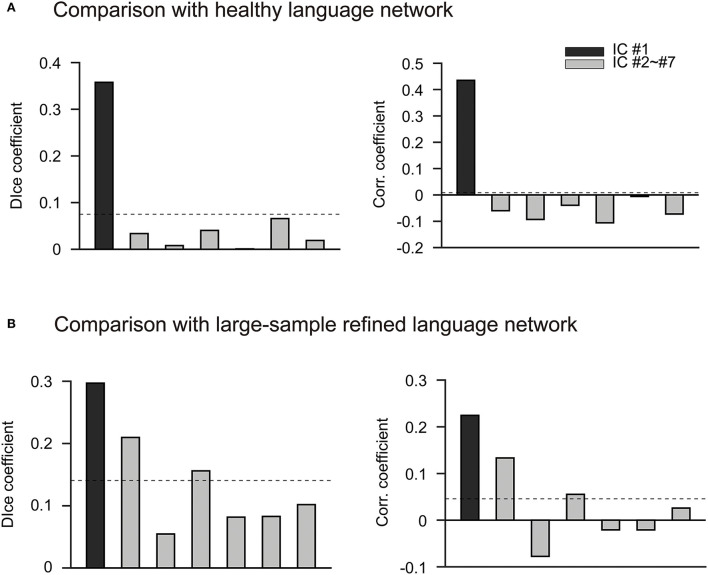
IC#1 has the most advantage in the spatial overlap and correlation with two well-established language networks. **(A)** Comparison with the parcellated language network in healthy people. Bar chart shows the comparison of seven ICs obtained by ICLM with the healthy language network defined in Yeo 17 networks. The Yeo 17 networks are parcellated from the resting-state imaging of healthy participants. The black bar indicates IC #1, and the gray bars indicate IC#2 to IC#7. The dashed lines in this figure represent the average correlation coefficient or dice coefficient across the seven ICs. Overall, IC#1 is significantly higher than the other ICs in terms of spatial overlap (left, DICE = 0.36) and correlation (right, *r* = 0.22) with the healthy language network. **(B)** Comparison with the large-sample refined language network. The comparison of the seven ICs obtained by ICLM with the language network from Neurosynth is shown. This target network is regarded as the “gold standard” since it is derived from the results of a meta-analysis of 1,101 language-related studies (www.neurosynth.org). The black bar indicates IC#1, and the gray bars indicate IC#2 to IC#7. The dashed lines in this figure represent the average correlation coefficient or dice coefficient across the seven ICs. Again, IC#1 exhibits the highest overlap (left, DICE = 0.3) and correlation (right, *r* = 0.44) with the “gold standard” language network, and therefore is defined as the specific lesioned language network in patients with aphasia. corr., correlation; IC, independent component; *r*, correlation coefficient.

We then performed the ICLM according to the flowchart shown in Supplementary Figure 2A. By combing all FC vectors that resulted from each patient's lesion mask, we got an FC matrix with a size of 49 × 32,894. The dimension of the signal subspace determined with the knee point method was 7 (Supplementary Figure 2B). We then performed an ICA on the signal subspace extracted by PCA to get ICs. We obtained seven spatially independent components by performing ICLM on the lesion maps from the 49 patients and normative fMRI data from the 50 HCs, as shown in Figure 2. To identify the language network from the seven ICs obtained by ICLM, we compared the pattern of ICs with the classic, healthy language network defined in the Yeo 17 networks parcellated from the resting-state imaging of healthy participants (Figure 3A), as well as with the large-sample refined language network as the “gold standard” (Figure 3B), which was derived from the meta-analysis results of language regions in 1,101 related studies using task-related imaging and patient data (www.neurosynth.org). Intriguingly, we found that IC#1 was outstandingly advantageous in the overlap (DICE = 0.36, compared with 0.08 as mean ICs) and correlation (*r* = 0.44, *p* < 0.001, significantly higher than the second strongest correlation, *Z* = 67.17, *p* < 0.001) with the healthy language network. Consistently, IC#1 was also highest in the overlap (DICE = 0.3, compared with 0.14 as mean ICs) and correlation (*r* = 0.22, *p* < 0.001, significantly higher than the second strongest correlation, *Z* = 12.05, *p* < 0.001) with the “gold standard” language network. Therefore, IC#1 was referred and defined as the specific lesioned language network in patients with aphasia.

The identified symptom-related language network were typically left-lateralized and included brain regions which are traditionally recognized as being involved in language processing, regions mostly distributed in the inferior frontal gyrus, superior frontal gyrus, and middle frontal gyrus, together with some other regions in premotor cortex. A complete list of MNI coordinates and *t* values for all statistically significant clusters is shown in Table 2. In addition, we found that the derived language network also revealed language-related regions in the cerebellum, which was consistent with previous findings (15, 55).

**Table 2:** ICLM results and MNI coordinates for the language network.

**Brain region**	**Brodmann area**	**Volume (cc)**		**Random effects: Max value (x, y, z)**		**MNI (x, y, z)**	
		**Left hemisphere**	**Right hemisphere**	**Left hemisphere**	**Right hemisphere**	**Left hemisphere**	**Right hemisphere**
Middle frontal gyrus	6, 8, 9, 10, 11, 46, 47	11.3	2.1	1.5 (−46, 6, 45)	1.3 (26, 43, −3)	(−46, 4, 49)	(26, 44, −1)
Precentral gyrus	6, 9, 44	0.5	/	1.5 (−50, 3, 49)	/	(−50, 1, 53)	
Sub-gyral	8, 21	0.4	4.5	1.2 (−14, 27, 42)	1.4 (22, 13, 26)	(−14, 26, 47)	(22, 12, 29)
Inferior frontal gyrus	9, 10, 11, 44, 45, 46, 47	10.4	3.6	1.4 (−61, 27, 3)	1.3 (53, 38, −11)	(−62, 28, 5)	(54, 40, −11)
Superior frontal gyrus	6, 8, 9, 10, 11	15.0	1.5	1.4 (−6, 30, 57)	1.4 (1, 28, 52)	(−6, 28, 64)	(1, 26, 58)
Medial frontal gyrus	6, 8, 9, 10, 11	4.0	0.8	1.3 (−10, 41, 39)	1.3 (1, 45, 42)	(−10, 40, 45)	(1, 44, 48)
Superior temporal gyrus	21, 22, 38, 39, 41, 42	2.2	2.3	1.3 (−33, 9, −40)	1.2 (38, −39, 7)	(−33, 11, −47)	(38, −40, 5)
Inferior temporal gyrus	20, 21	2.7	/	1.3 (−50, −21, −28)	/	(−51, −20, −35)	/
Inferior parietal lobule	39, 40	0.5	/	1.2 (−66, −41, 26)	/	(−67, −44, 26)	/
Middle temporal gyrus	20, 21, 22, 38	4.1	2.0	1.2 (−43, 6, −36)	1.2 (38, −41, 7)	(−43, 8, −43)	(38, −43, 5)
Fusiform gyrus	18, 19, 20	0.9	0.1	1.2 (−53, −9, −26)	1.1 (25, −74, −12)	(−54, −8, −31)	(25, −76, −19)
Supramarginal gyrus	39, 40	1.5	/	1.2 (−63, −49, 29)	1.1 (−57, −64, 31)	(−64, −52, 29)	(−58, −67, 30)
Rectal gyrus	11	0.6	0.4	1.1 (−2, 30, −24)	1.2 (4, 49, −25)	(−2, 32, −27)	(4, 52, −27)
Orbital gyrus	11	0.2	0.2	1.1 (−6, 38, −21)	1.2 (5, 47, −27)	(−6, 40, −23)	(5, 50, −29)
Angular gyrus	39	0.1	/	1.1 (−54, −56, 36)	/	(−55, −59, 36)	/
Uncus	20, 38	0.1	/	1.0 (−29, 3, −40)	/	(−29, 5, −47)	/

ICLM, independent component-based lesion mapping; MNI, Montreal Neurological Institute; cc, cubic centimeters; Max, maximum.

### High specificity of the lesioned language network in detecting behavioral dysfunction

**Figure 4:**
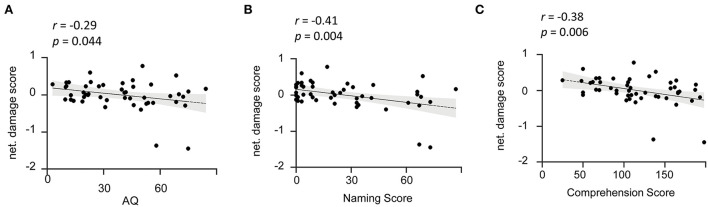
Damage level of the identified language network is reversely associated with aphasia severity. **(A)** Network damage level is associated with AQ. The scatter plot illustrates a significant negative correlation (*r* = −0.29, *p* = 0.044) between network damage scores of the identified language network and patients' AQ. **(B,C)** Network damage level is associated with the naming and comprehension subtests. The scatter plots show that the language network damage score is negatively correlated with naming (*r* = −0.41, *p* = 0.004) and comprehension (*r* = −0.38, *p* =0.006) scores. AQ, Aphasia Quotient. The solid line represents the best linear fit with its 95% confidence interval (shaded area). Black dots are individual data points.

To further investigate the role of the lesioned language network in aphasia symptoms, we calculated the correlation between the network damage score calculated within the lesioned language network and the AQ performance of the patients. As a result, we found a significant negative correlation of damage level with the total score of AQ (*r* = −0.29, *p* = 0.04) (Figure 4A). In addition, the correlation coefficients between the language network damage score and the subtest scores were also calculated. We found that the language network damage scores were also negatively correlated with the naming scores (*r* = −0.41, *p* < 0.001) and comprehension (*r* = −0.38, *p* = 0.01) (Figures 4B,C and Supplementary Figure 3). Accordingly, the lesioned language network was significantly associated with lesion-induced language deficits. The results indicate that the more severe the damage within the derived language network of patients with post-stroke aphasia, the lower the patient's AQ, that is, the more severe the aphasia (40). We thus succeeded in mapping the symptom-related language network associated with aphasia severity in patients.

In comparison, we conducted an additional correlation analysis of network damage score in the other six ICs with the AQ performance (Supplementary Figure 4). None of the ICs were found to be responsible for the behavioral dysfunction (*p* > 0.05 for each of the six ICs), indicating the specificity of lesioned language network in causing aphasia symptoms. To further prove the efficiency and accuracy of the language network derived from our ICLM method, we also compared it with the healthy language network as well as the refined language network (Supplementary Figure 5). As a result, we found no significance of the correlation between the damage score in the healthy language network and the language dysfunctions in patients (*r* = 0.17, *p* = 0.26). Such relationship was significantly weaker when compared with the association of our lesioned language network and symptoms (*Z* = −2.11, *p* = 0.034 in comparing the two correlation coefficients). Together, the result of the traditional parcellated language network indicated a low specificity when we applied the network derived from the healthy participants to the lesioned patients. Moreover, the damage score calculated in the “golden standard” language network has a strong association with behavioral abnormality (*r* = −0.41, *p* = 0.004). The association to behavior from our lesioned language network turned out to quite resemble the result calculated from large-sampled refined language network (Z = −0.82, *p* = 0.41). Given the authority of the refined language network, which was extracted from thousands of language-related research studies, our lesioned language network derived from only 49 patients exhibited a high efficiency in detecting the symptom-related language map.

### Reproducibility and stability analysis

**Figure 5:**
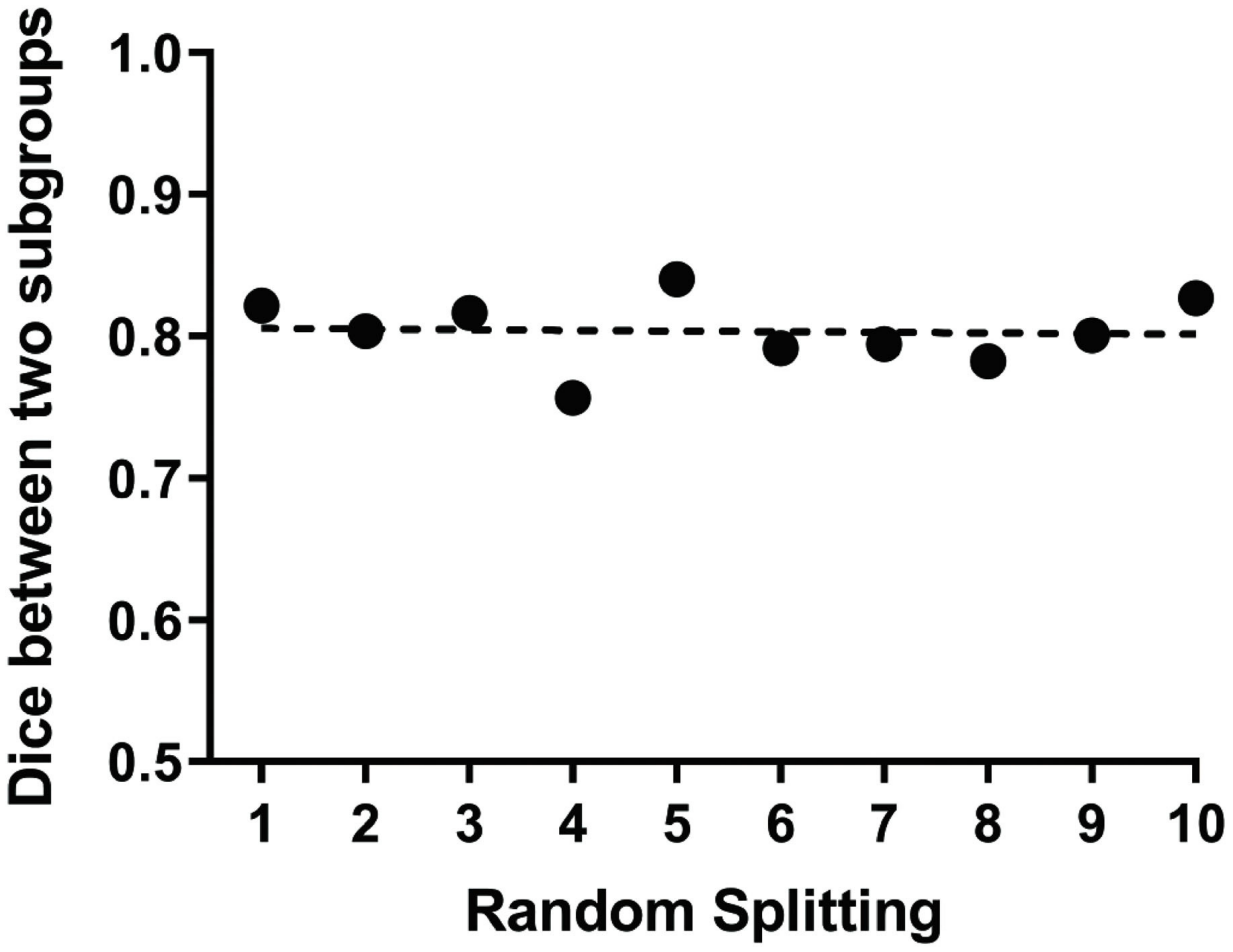
ICLM of randomly split subgroups reveals adequate reproducibility of this method. The scatter plot shows the dice values between the language networks of the two subgroups extracted in 10 different runs. Each point represents the dice value between two language networks generated by ICLM within two randomly split subgroups. The dotted line indicates the mean value of 10 dice values, which is 0.8, demonstrating the relatively strong reproducibility of ICLM.

We further tested the reproducibility and stability of the ICLM method in yielding the language network from patients with aphasia. To exclude the possible confounds of distinct neurofunctional mechanisms taking place in different patients, we conducted a control analysis on the different subgroups of patients either by random splitting or according to clinical features. In the former method, we repeated the calculation of the pairwise correlation between the language networks of the two randomly divided subgroups extracted in 10 different runs and obtained an average dice count of 0.8. The pairwise dice from the different runs is shown in the graph (Figure 5). This demonstrates that the language network obtained with the ICLM method was highly reproducible. In the latter method, we grouped the patients according to their lesion types (ischemic and hemorrhagic strokes), disease stages (acute/subacute and chronic stokes), or symptom severity (stroke with minor and severe aphasia). The identified language networks with the ICLM method exhibited a spatial distribution quite similar to the one using all the patients (Supplementary Figure 6).

To validate the stability in generating the lesioned language network with the ICLM method, we also changed the sample size of the healthy cohorts in constructing FC matrices as well as the threshold in defining the lesioned language network. Both the identification of the lesioned language network and the correlation with behavioral dysfunction were unchanged by repeating the ICLM with the different parameters (Supplementary Figures 7A,B). Our results thus proved the robustness of ICLM by including different healthy populations as well as changing the network threshold.

## Discussion

In this study, we identified a language network from mixed lesion-affected brain regions in patients with moderate to severe post-stroke aphasia by ICLM. We found that the language network damage scores were negatively correlated with AQ and the other two subtests, naming and auditory comprehension, which verified the identified language circuit. By detecting a specific lesioned language network that otherwise could not be identified with the traditional VLSM and LNM methods, our ICLM method succeeded in mapping the language network in relation to clinical behavioral deficits in post-stroke aphasia. The highlight of this study also lies in the high specificity of the lesioned language network in associating the behavioral dysfunction, which was more superior to the classic parcellated language network and as good as the large-sample refined language network.

Based on the improved ICLM method, this study mapped the language network in post-stroke aphasia. The large-scale network was mainly distributed in the temporal lobe, prefrontal lobe, parietal cortex, and cerebellum, which generally covers the circuits of speech and semantic processing. This is basically consistent with previous research findings. For example, our language circuit includes two key nodes of the brain language network, the posterior middle temporal gyrus and the inferior frontal gyrus (56). The resulting circuit also includes the angular gyrus, which is related to semantic fluency (57), the middle temporal gyrus, which is related to speech processing, and the superior temporal gyrus, which is involved in the semantic-phonological interaction process (58). These functions are highly related to naming and comprehension ability, which corresponds to the two significantly correlated WAB subtests. Furthermore, we found that the derived language network also discovered language-related maps in the cerebellum, which fitted well with previous findings (15).

Compared with those of the traditional VLSM and LNM methods, our results have shown that lesion mapping incorporating ICA can accurately identify language regions from multiple lesion-affected networks. The excellent performance of our method benefited from the following reasons.

First, ICLM mapped the lesion-affected networks more independently from the healthy controls instead of directly using fMRI signals from the patients. This is because the brain tissue at the lesion location in patients with stroke is mostly destroyed, and functional neural activities within or near the lesion site can no longer accurately reflect the normal physiology or connectivity of ROIs. Also, the abnormalities in BOLD signals detected in patients with post-stroke are not only affected by the focal lesion but may also be affected by secondary compensation or adaptive reorganization of brain networks away from the lesioned area. Therefore, our method uses an indirect functional connectivity profile from a large cohort of healthy participants as in LNM, which can map brain regions that are normally associated with lesioned sites. The complementary application of LM and indirect fMRI in the future may bring some new insights into the field of neurology (15, 59).

Second, our method differs from the conventional LNM in that the introduced ICA makes it more suitable for mapping the symptom-related network from complicated lesions. Conventional LNM directly applies lesions as seed regions to calculate FC and then simply overlaps the maps obtained from each patient to identify common brain regions. However, when the stroke lesion is heterogeneously distributed, the lesion-related brain regions calculated by traditional LNM may also exhibit large inter-subject variability, leading to a question whether meaningful information could be fully obtained from the plain overlapping approach. The mapping results of conventional LNM confirmed our speculation, with very limited common regions found and failed to establish a relationship with aphasia symptom. In comparison, ICLM utilizes the full set of lesion-affected connectivity information and leverages ICA to isolate the symptom-related component from mixed signals. As a blind source separation technique, ICA has been commonly conducted in brain network analysis of cerebrovascular diseases in recent years. When both the source signal and the mixing matrix are unknown, it can find out mutually independent implicit components by analyzing the high-order correlation between multidimensional observational data and complete the extraction of the independent source signal (60–62). After calculating the lesion-affected FC, we performed an ICA to extract independent components from the combined FC matrix from all the patients and identified the language network that is linked to the aphasia-related symptoms. Through this procedure, ICLM can discriminate the single concerned network from a mixed bag of lesion-affected networks, of which the results were robust after reproducibility analysis.

Third, our definition of network damage score differs from that of previous LNM studies. Conventional LNM has been applied to studies on memory and depressive disorders and successfully obtained memory and depressive circuits (14, 49). These studies found that network damage scores were associated with depression severity and memory behavioral scores. The network damage score in these studies was calculated by summing the *t*-values for each voxel that fell within the intersection of the derived circuit and the lesion mask. However, summed *t*-values are heavily dependent on the number of damaged voxels when calculating the correlation between the network damage score and behavior scores, so this index is highly perturbed by the size of the lesions. In contrast, the network damage score in this study was replaced with the max of *z*-values of the IC map that fell within the intersection of the derived language network and the lesion mask for each patient (63). In large lesion studies, such a network damage score definition may better represent the extent to which the lesion and language network intersect.

As a result, we identified the lesioned language network extracted with the ICLM method. The symptom-related language network turned out to be mostly distributed in the inferior frontal gyrus (IFG), superior frontal gyrus (SFG), superior temporal gyrus (STG), and middle frontal gyrus (MFG) in the left hemisphere, which were traditionally recognized as key regions in healthy language processing (64). Specifically, the opercular and triangular part of the IFG was traditionally known as Broca's area (BA), and the posterior aspect of the STG was traditionally known as Wernicke's area (WA). Besides, a number of regions we found were covered in the premotor cortex (Brodmann's area 6), which was referred to as language supplementary motor area (SMA). Compared with previous classical language networks, our lesioned language network was also more specific to these key regions that played crucial roles in clinical practice and for localization in treatment planning (38, 65). For instance, a body of research has indicated a strong increase of activation in SFG and a high correlation with language recovery in patients with post-stroke. The IFG, SMA, BA, and WA were also used as targets of non-invasive brain stimulation to facilitate language rehabilitation (66, 67). Therefore, our lesioned language network showed a high specificity in identifying the crucial regions responsible for behavior deficits.

Furthermore, the symptom-related brain circuits identified by ICLM were not only specific to the behavioral deficit but also showed relatively high reproducibility and stability. Among the seven ICs segregated by ICA, only one was significantly correlated with the patients' language assessments, indicating the high specificity of our method. In other words, we have established an association between the network damage score and the aphasia severity assessment exclusively in one IC, namely, the language network. The estimation power of our lesioned language network was significantly higher than that of the classical healthy language network and approximated to the meta-analysis results from thousands of language-related research studies, indicating the reliable symptom-localization ability of ICLM. Further with the reproducibility analysis, we found that the determined symptom-related regions were highly similar between every two independent samples and different types of subgroups, which again showed the great reliability of our method.

This upgraded, accurate, and reliable lesion mapping method can be beneficial to clinical practices. The precise delineation of a symptom-related functional network has direct clinical implications for optimizing patient rehabilitation strategies. Neuromodulation techniques such as transcranial magnetic stimulation can promote rehabilitation by modulation of targeted brain regions or of their corresponding functional connectivity (66). The clear symptom-biomarker mapped by ICLM could facilitate patient management with more targeted, effective, and optimal intervention planning. Furthermore, ICLM can also be performed to map other symptom-related functional networks besides language. Given that neural diseases with complex lesion topographies are probably common in patients, this method has a great potential in helping discover the neural correlates of multiple functional deficits. Finally, although the regional damage level was correlated with overall or partial aphasia severity, whether the feature of this network can serve as a predictive marker of behavioral improvement remains to be determined. In future studies, we can further validate the prognostic value of the obtained functional network.

There are still some limitations in this study. First of all, the sample size of this study is relatively small. In the future, we should try to apply this method to a larger database to observe its robustness. Second, we have only performed ICLM on post-stroke aphasia and cannot verify whether this method can be generalized to different clinical syndromes. This method should be also applied to other diseases in the future to evaluate its sensitivity and specificity. Third, we mainly focused on the language network of the cerebral cortex and did not further explore the circuits of white matter areas such as fiber tracts. Finally, ICLM may also need to consider many covariates such as age, gender, and education. The healthy control group was young, and age may affect functional connectivity strength (68). However, previous studies applying conventional LNM to the elderly have not concluded that age directly changed results, so this should not affect our interpretation of ICLM (15).

In conclusion, this study mapped a specific language network in post-stroke aphasia with high heterogeneous regions and linked the lesioned language network to aphasia severity. We consider ICLM to be an important complement to lesion behavior mapping by improving the precise network localization of complicated lesions. In the future, ICLM may be combined with non-invasive brain stimulation to identify key stimulation targets for specific symptoms and promote functional recovery in complicated brain diseases.

## Data availability statement

The raw data supporting the conclusions of this article will be made available by the authors, without undue reservation.

## Ethics statement

The studies involving human participants were reviewed and approved by the Ethics Committee of the China Rehabilitation Research Center. The patients/participants provided their written informed consent to participate in this study.

## Author contributions

WR and HZ designed the experiment. WR, CJ, and JZ executed the experiment. WR, CJ, BW, WY, and JZ performed the analysis of the data. WR, CJ, SL, and YH produced the manuscript figures and tables. WR, YZ, and HZ wrote the manuscript. All authors reviewed the manuscript, contributed to the article, and approved the submitted version.

## Funding

This research was funded by the Research Project on Joint Training of Doctors by University of Health and Rehabilitation Sciences and China Rehabilitation Research Center (Grant No. 2020kfdx-005). It was also funded by the Scientific Research Project of China Rehabilitation Research Center (Grant No. 2019zx-02).

## Conflict of interest

The authors declare that the research was conducted in the absence of any commercial or financial relationships that could be construed as a potential conflict of interest.

## Publisher's note

All claims expressed in this article are solely those of the authors and do not necessarily represent those of their affiliated organizations, or those of the publisher, the editors and the reviewers. Any product that may be evaluated in this article, or claim that may be made by its manufacturer, is not guaranteed or endorsed by the publisher.
