# Supplementary material for: A precise language network revealed by the independent component-based lesion mapping in post-stroke aphasia
Source: Front Neurol. 2022 Sep 30;13:981653. doi: 10.3389/fneur.2022.981653 (PMC9561861; doi:10.3389/fneur.2022.981653)
Supplement: Supplementary file 1 [file Presentation_1.PPTX]

## Slide 1
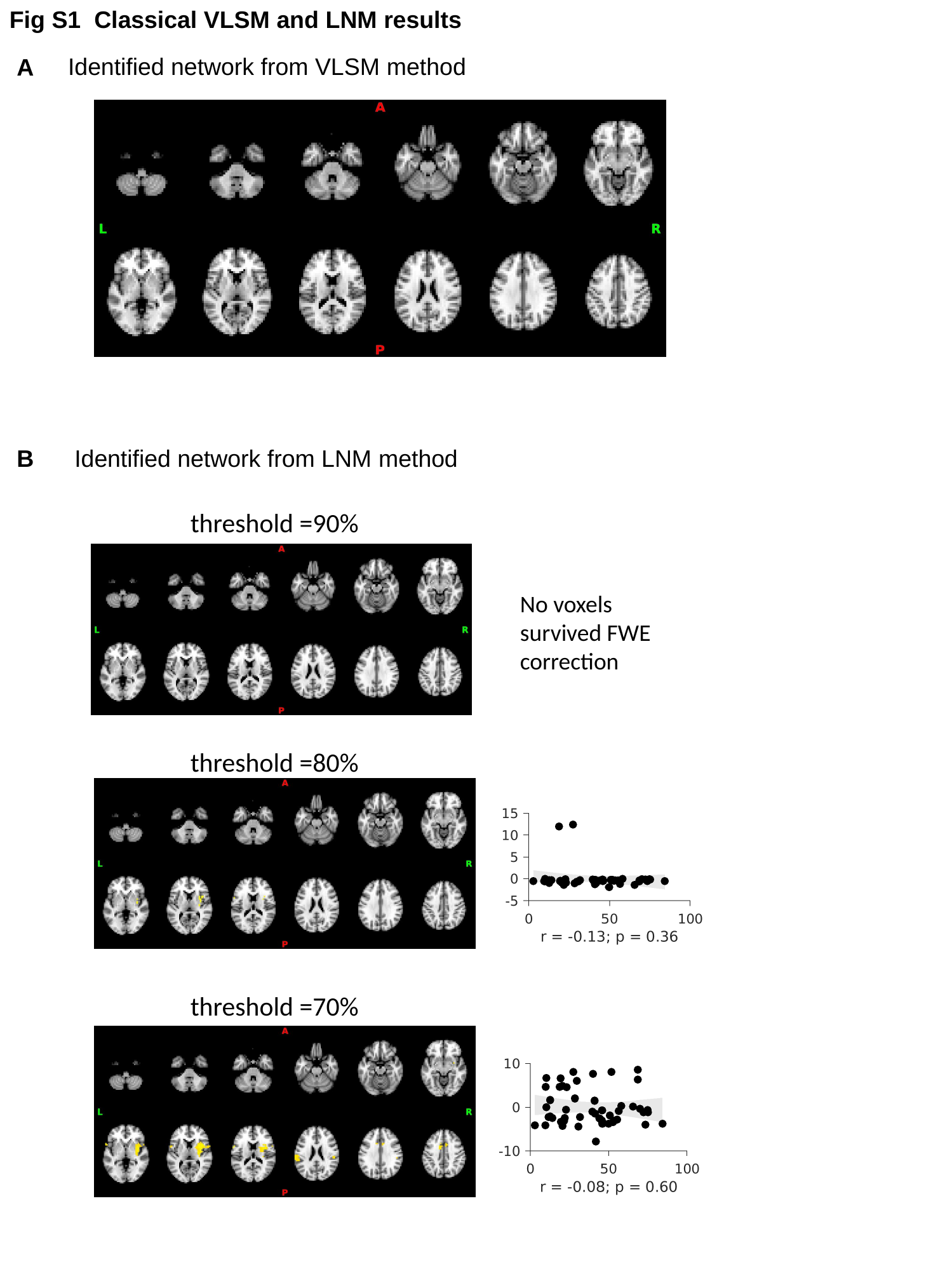

Fig S1 Classical VLSM and LNM results
Identified network from VLSM method
A
B
Identified network from LNM method
threshold =90%
No voxels survived FWE correction
threshold =80%
threshold =70%

## Slide 2
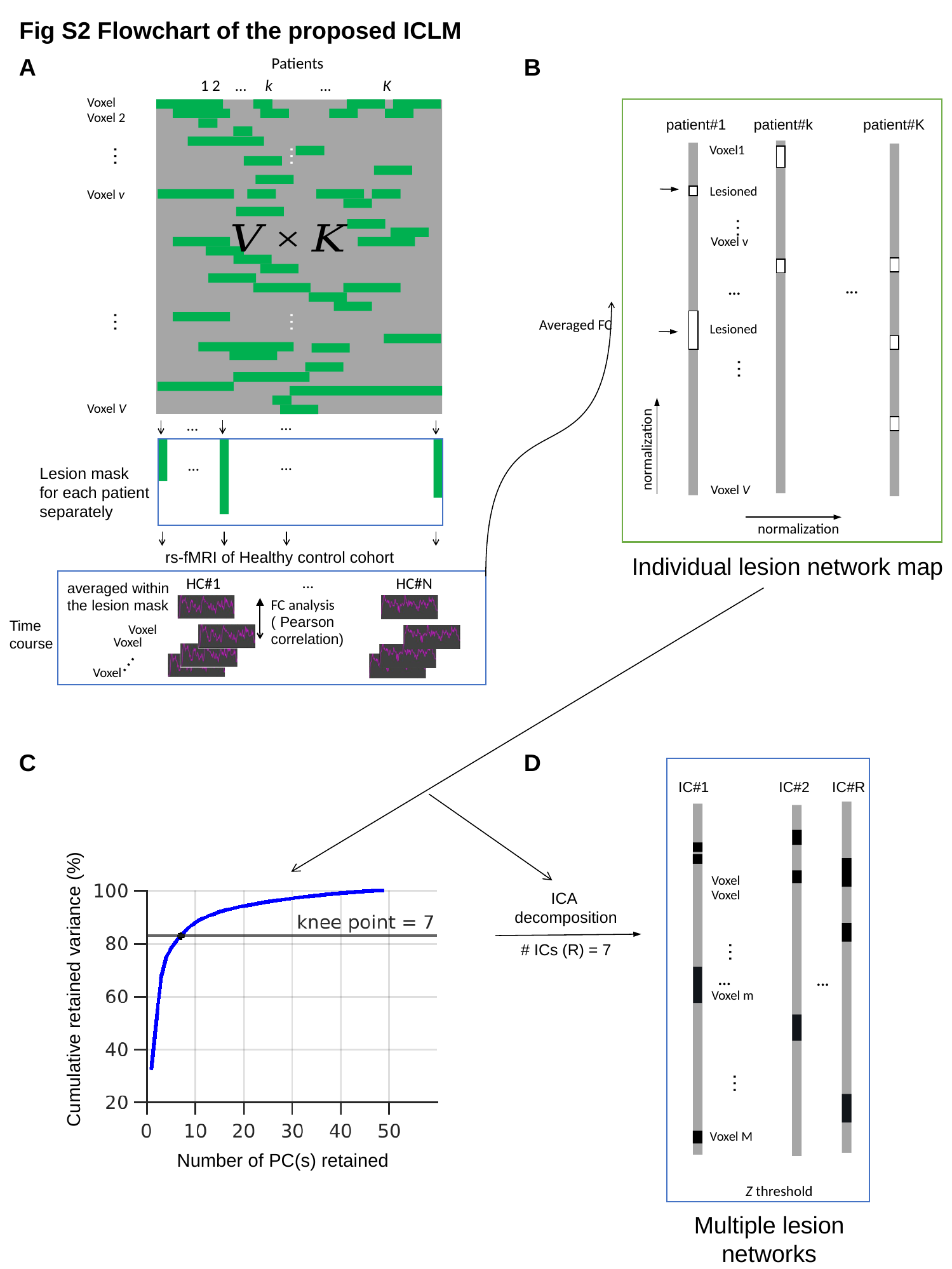

Fig S2 Flowchart of the proposed ICLM
A
B
Patients
1 2 ... k ... K
Voxel 2
patient#1
patient#k
patient#K
Voxel1
…
…
Lesioned
Voxel v
…
Voxel v
...
...
Averaged FC
…
…
Lesioned
…
Voxel V
...
...
normalization
...
...
Lesion mask
for each patient
separately
Voxel V
normalization
rs-fMRI of Healthy control cohort
Individual lesion network map
...
HC#1
HC#N
averaged within the lesion mask
FC analysis
( Pearson correlation)
Time course
…
C
D
IC#1
IC#2
IC#R
ICA
decomposition
# ICs (R) = 7
…
...
...
Cumulative retained variance (%)
Voxel m
…
Voxel M
Number of PC(s) retained
Z threshold
Multiple lesion networks

## Slide 3
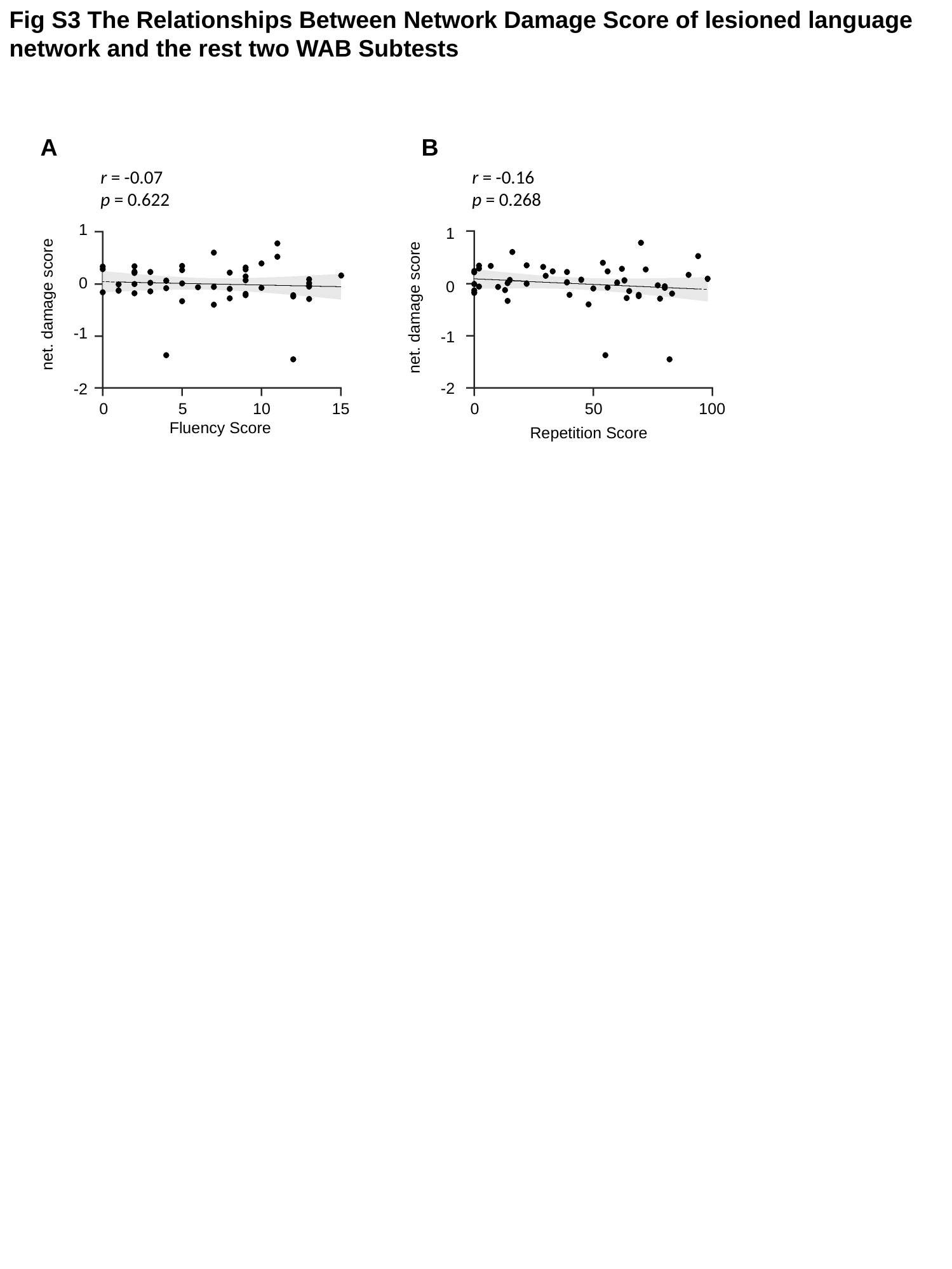

Fig S3 The Relationships Between Network Damage Score of lesioned language network and the rest two WAB Subtests
A
B
r = -0.07
p = 0.622
r = -0.16
p = 0.268
1
1
0
0
net. damage score
net. damage score
-1
-1
-2
-2
0
50
100
0
5
10
15
Fluency Score
Repetition Score

## Slide 4
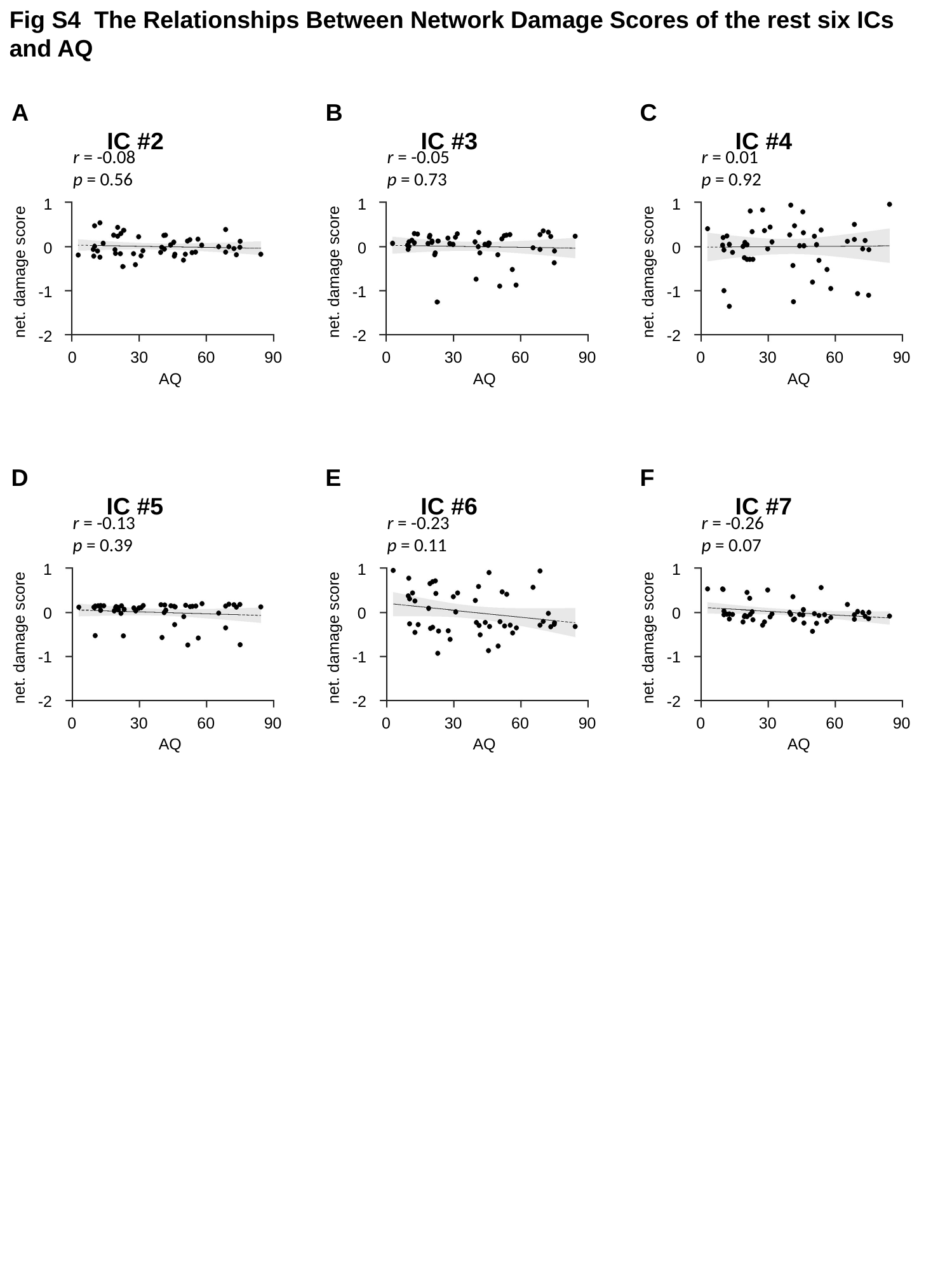

Fig S4 The Relationships Between Network Damage Scores of the rest six ICs and AQ
B			IC #3
r = -0.05
p = 0.73
1
0
net. damage score
-1
-2
0
30
60
90
AQ
C			IC #4
r = 0.01
p = 0.92
1
0
net. damage score
-1
-2
0
30
60
90
AQ
A			IC #2
r = -0.08
p = 0.56
1
0
net. damage score
-1
-2
0
30
60
90
AQ
D			IC #5
r = -0.13
p = 0.39
1
0
net. damage score
-1
-2
0
30
60
90
AQ
E			IC #6
r = -0.23
p = 0.11
1
0
net. damage score
-1
-2
0
30
60
90
AQ
F			IC #7
r = -0.26
p = 0.07
1
0
net. damage score
-1
-2
0
30
60
90
AQ

## Slide 5
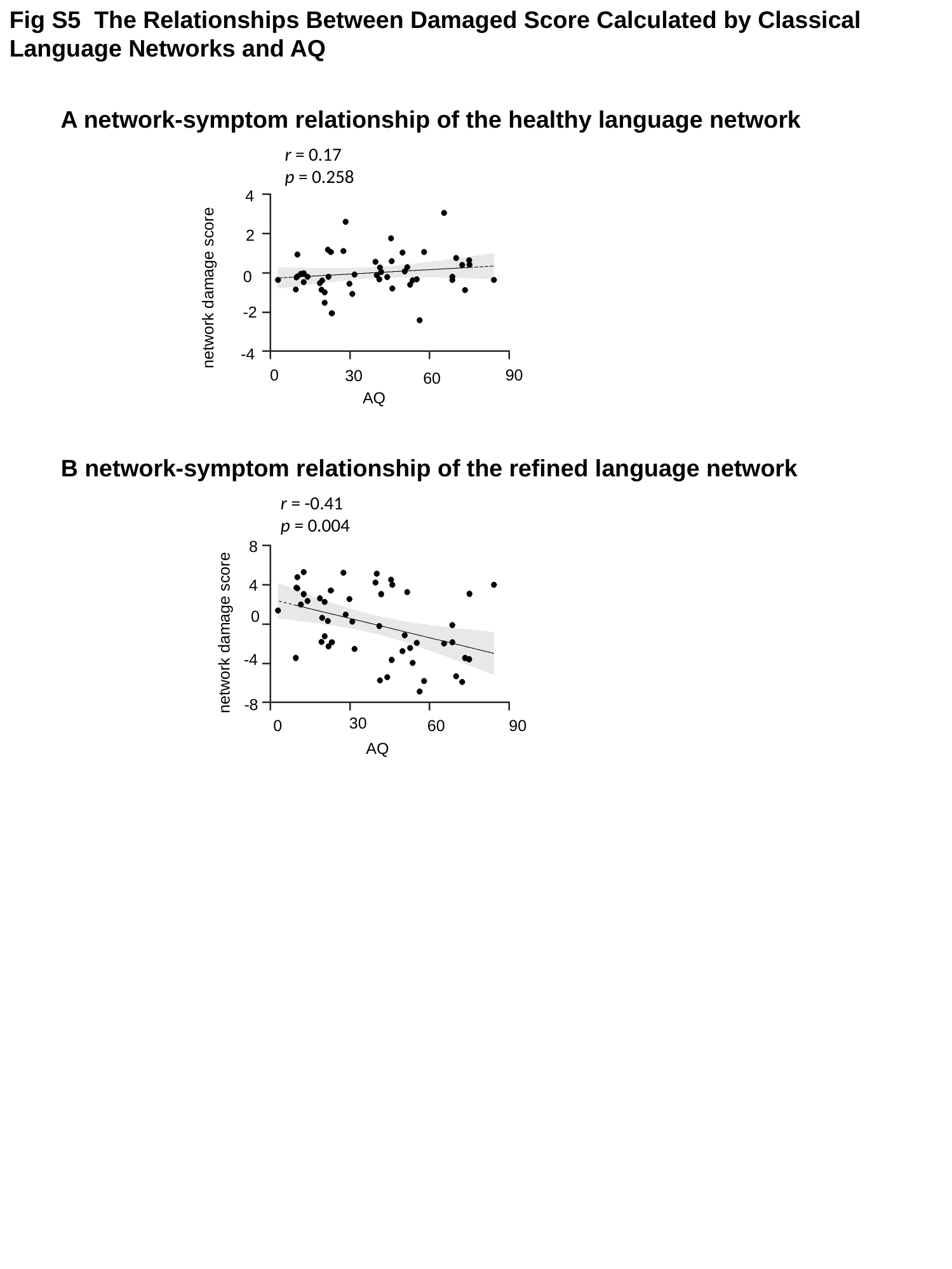

Fig S5 The Relationships Between Damaged Score Calculated by Classical Language Networks and AQ
A network-symptom relationship of the healthy language network
r = 0.17
p = 0.258
4
2
network damage score
0
-2
-4
0
90
30
60
AQ
B network-symptom relationship of the refined language network
r = -0.41
p = 0.004
8
4
0
network damage score
-4
-8
30
0
60
90
AQ

## Slide 6
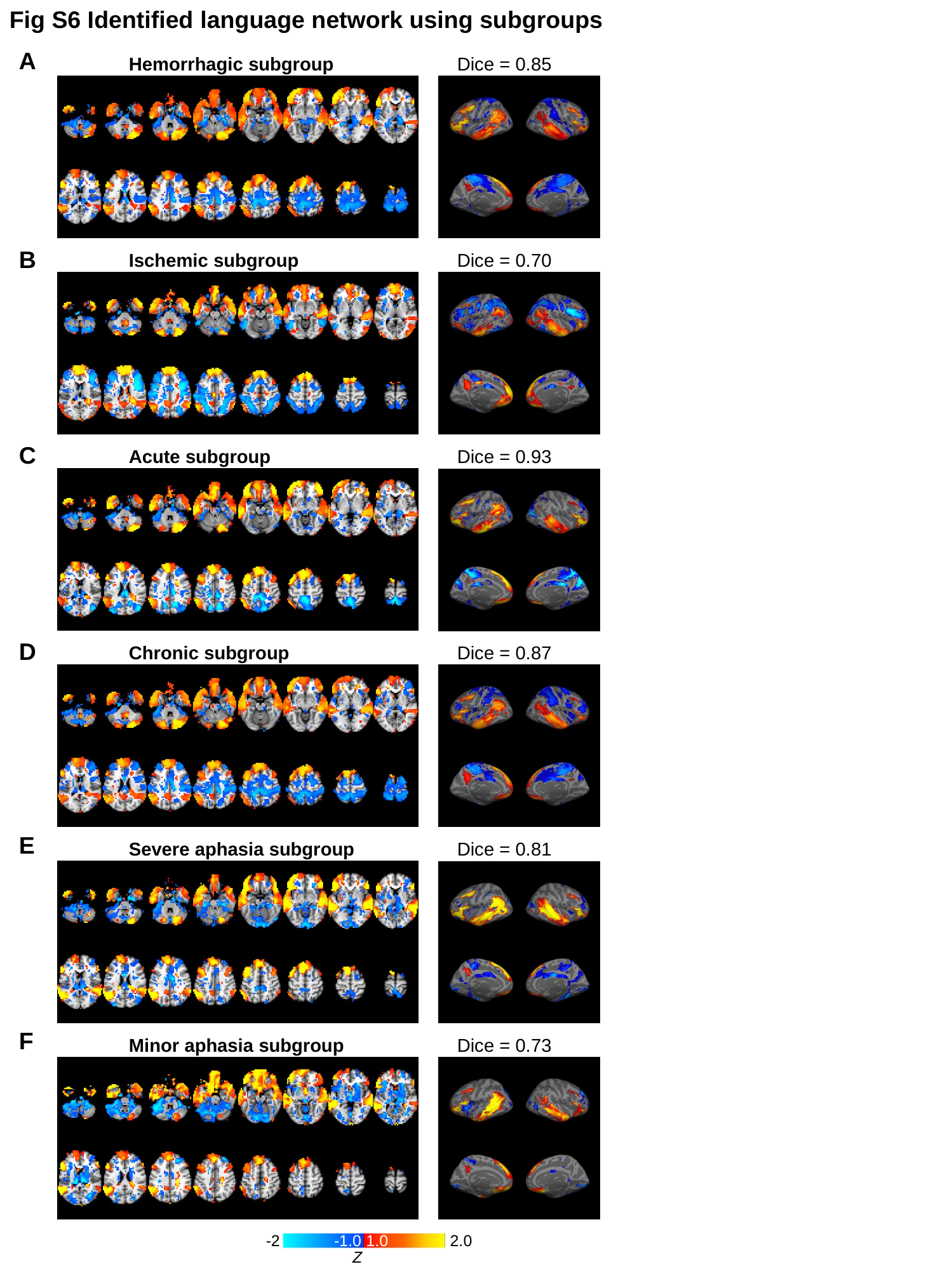

Fig S6 Identified language network using subgroups
A
Hemorrhagic subgroup
Dice = 0.85
B
Ischemic subgroup
Dice = 0.70
C
Acute subgroup
Dice = 0.93
D
Chronic subgroup
Dice = 0.87
E
Severe aphasia subgroup
Dice = 0.81
F
Minor aphasia subgroup
Dice = 0.73
-1.0
-2
1.0
2.0
Z

## Slide 7
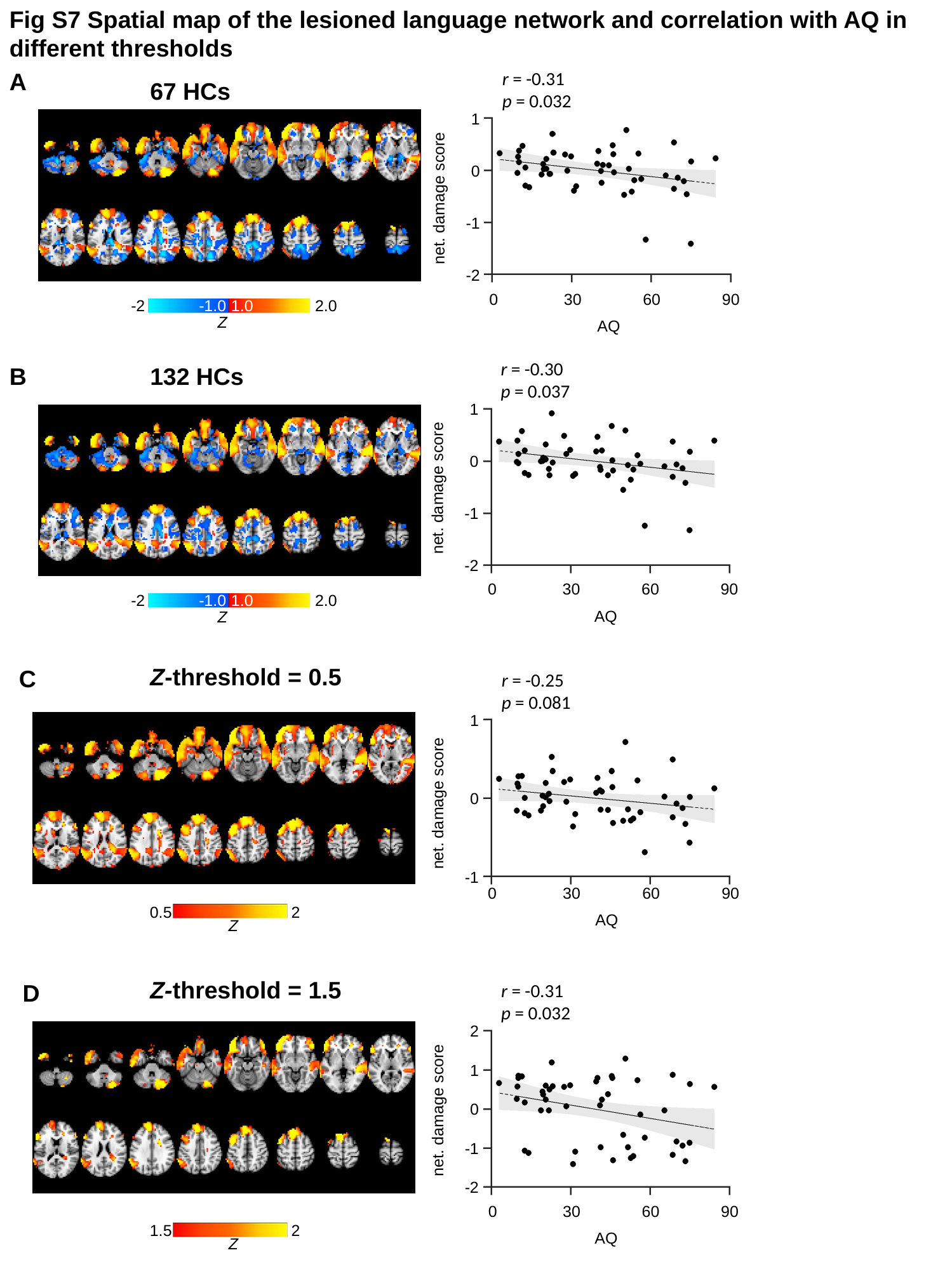

Fig S7 Spatial map of the lesioned language network and correlation with AQ in different thresholds
A
r = -0.31
p = 0.032
67 HCs
1
0
net. damage score
-1
-2
-1.0
-2
1.0
2.0
Z
0
30
60
90
AQ
r = -0.30
p = 0.037
B
132 HCs
1
0
net. damage score
-1
-2
0
30
60
90
-1.0
-2
1.0
2.0
Z
AQ
Z-threshold = 0.5
C
r = -0.25
p = 0.081
1
0
net. damage score
-1
0
30
60
90
0.5
2
Z
AQ
Z-threshold = 1.5
D
r = -0.31
p = 0.032
2
1
0
net. damage score
-1
-2
0
30
60
90
1.5
2
Z
AQ

## Slide 8
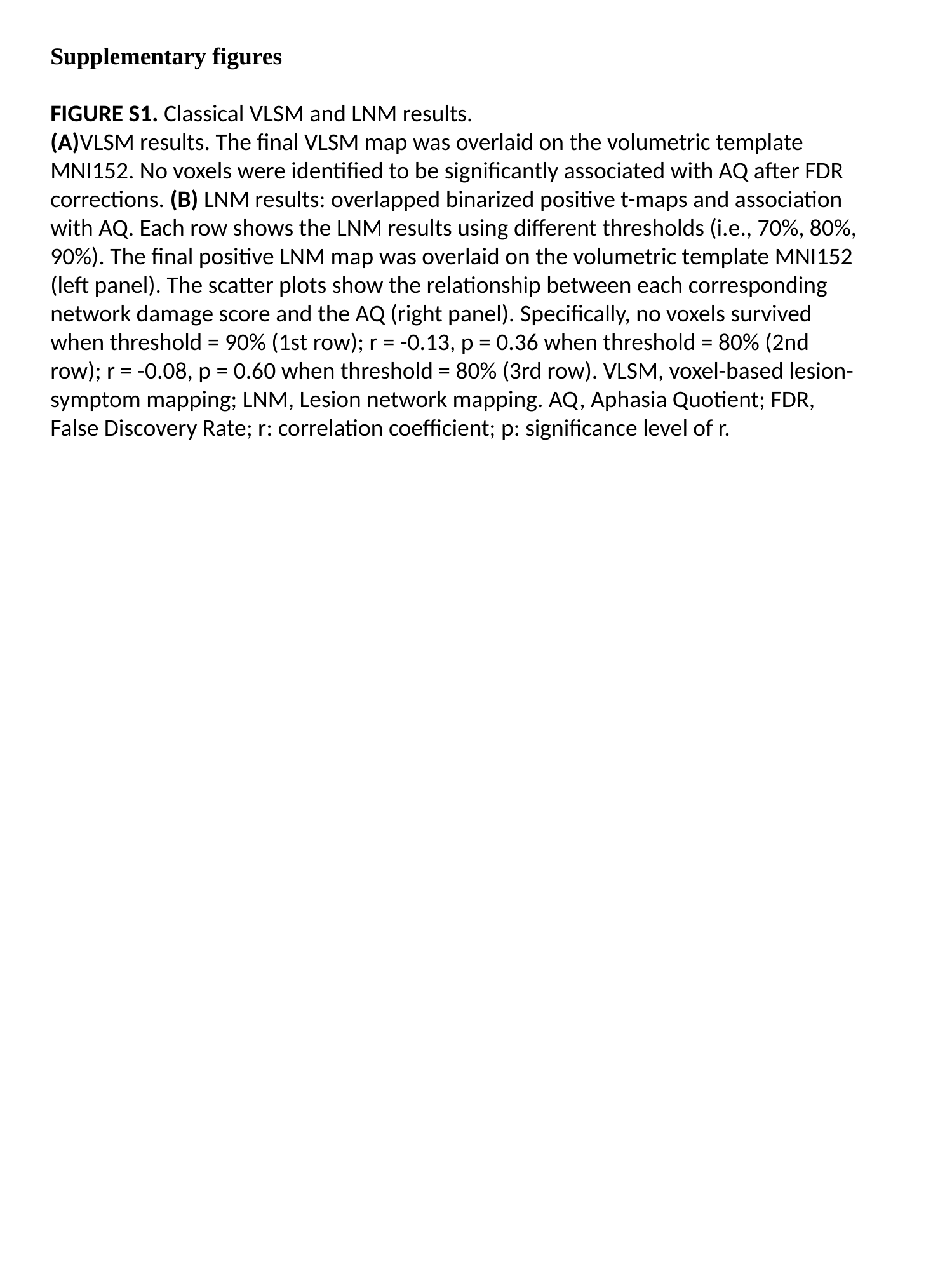

Supplementary figures
FIGURE S1. Classical VLSM and LNM results.
(A)VLSM results. The final VLSM map was overlaid on the volumetric template MNI152. No voxels were identified to be significantly associated with AQ after FDR corrections. (B) LNM results: overlapped binarized positive t-maps and association with AQ. Each row shows the LNM results using different thresholds (i.e., 70%, 80%, 90%). The final positive LNM map was overlaid on the volumetric template MNI152 (left panel). The scatter plots show the relationship between each corresponding network damage score and the AQ (right panel). Specifically, no voxels survived when threshold = 90% (1st row); r = -0.13, p = 0.36 when threshold = 80% (2nd row); r = -0.08, p = 0.60 when threshold = 80% (3rd row). VLSM, voxel-based lesion-symptom mapping; LNM, Lesion network mapping. AQ, Aphasia Quotient; FDR, False Discovery Rate; r: correlation coefficient; p: significance level of r.

## Slide 9
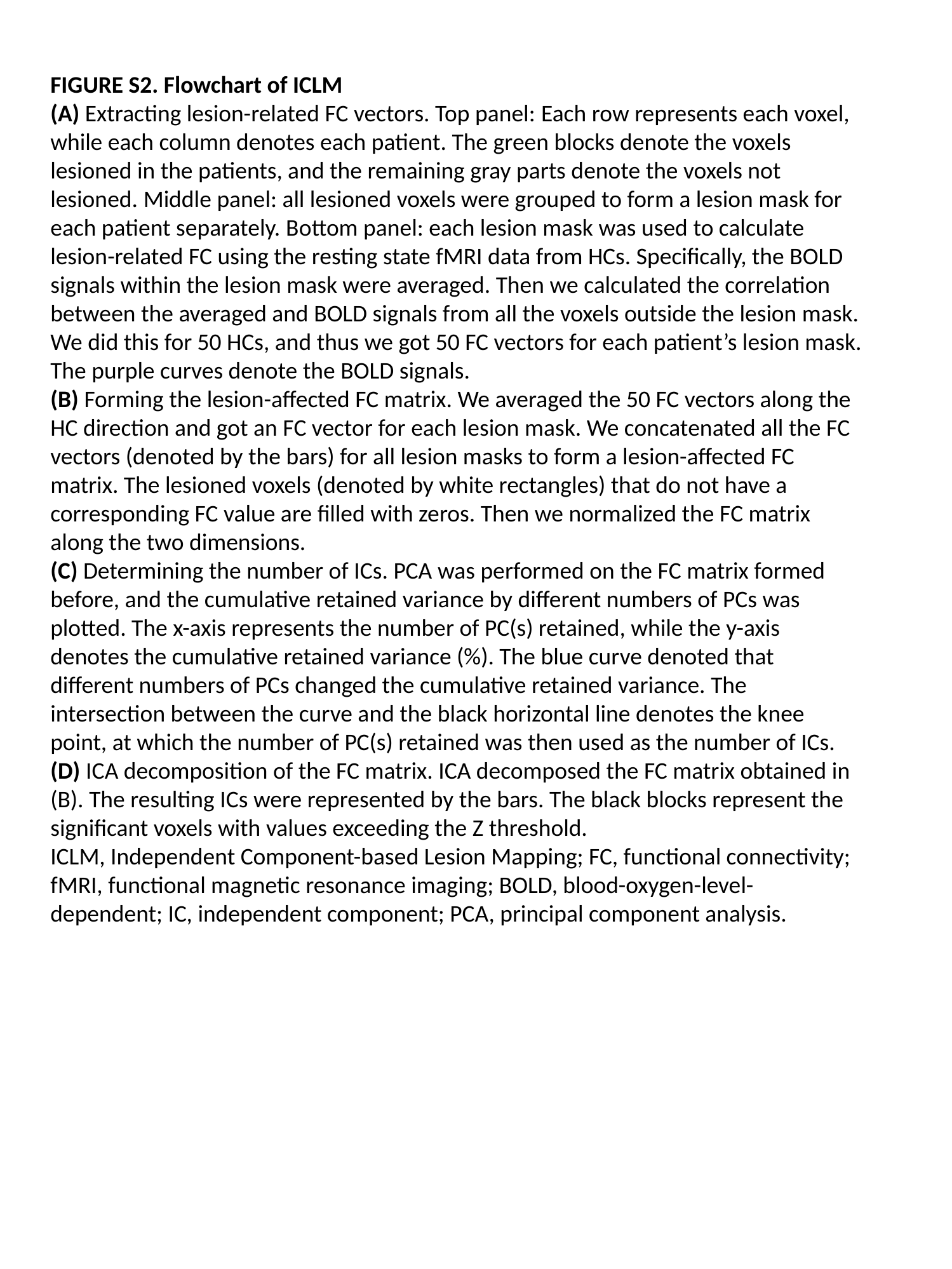

FIGURE S2. Flowchart of ICLM
(A) Extracting lesion-related FC vectors. Top panel: Each row represents each voxel, while each column denotes each patient. The green blocks denote the voxels lesioned in the patients, and the remaining gray parts denote the voxels not lesioned. Middle panel: all lesioned voxels were grouped to form a lesion mask for each patient separately. Bottom panel: each lesion mask was used to calculate lesion-related FC using the resting state fMRI data from HCs. Specifically, the BOLD signals within the lesion mask were averaged. Then we calculated the correlation between the averaged and BOLD signals from all the voxels outside the lesion mask. We did this for 50 HCs, and thus we got 50 FC vectors for each patient’s lesion mask. The purple curves denote the BOLD signals.
(B) Forming the lesion-affected FC matrix. We averaged the 50 FC vectors along the HC direction and got an FC vector for each lesion mask. We concatenated all the FC vectors (denoted by the bars) for all lesion masks to form a lesion-affected FC matrix. The lesioned voxels (denoted by white rectangles) that do not have a corresponding FC value are filled with zeros. Then we normalized the FC matrix along the two dimensions.
(C) Determining the number of ICs. PCA was performed on the FC matrix formed before, and the cumulative retained variance by different numbers of PCs was plotted. The x-axis represents the number of PC(s) retained, while the y-axis denotes the cumulative retained variance (%). The blue curve denoted that different numbers of PCs changed the cumulative retained variance. The intersection between the curve and the black horizontal line denotes the knee point, at which the number of PC(s) retained was then used as the number of ICs.
(D) ICA decomposition of the FC matrix. ICA decomposed the FC matrix obtained in (B). The resulting ICs were represented by the bars. The black blocks represent the significant voxels with values exceeding the Z threshold.
ICLM, Independent Component-based Lesion Mapping; FC, functional connectivity; fMRI, functional magnetic resonance imaging; BOLD, blood-oxygen-level-dependent; IC, independent component; PCA, principal component analysis.

## Slide 10
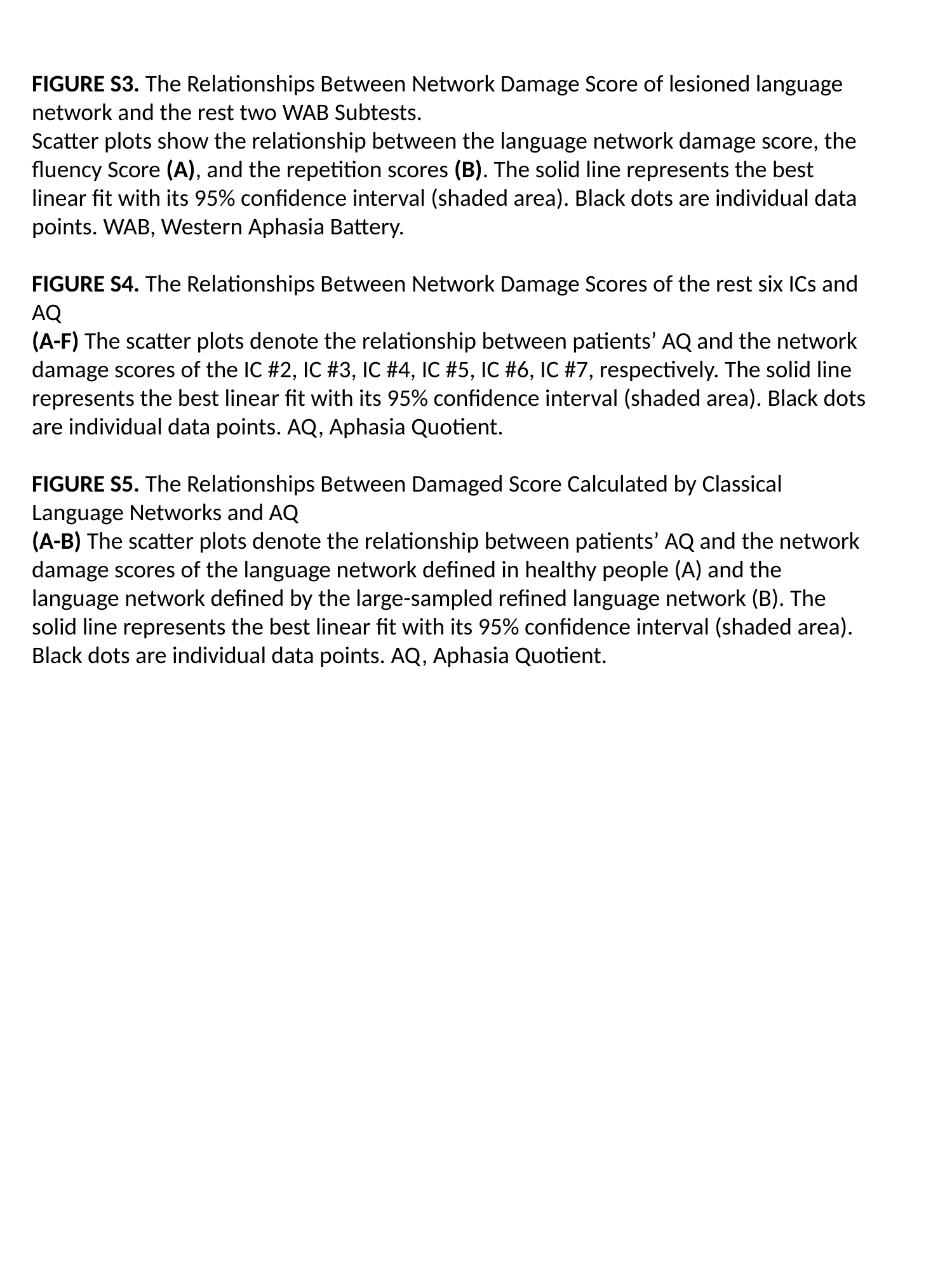

FIGURE S3. The Relationships Between Network Damage Score of lesioned language network and the rest two WAB Subtests.
Scatter plots show the relationship between the language network damage score, the fluency Score (A), and the repetition scores (B). The solid line represents the best linear fit with its 95% confidence interval (shaded area). Black dots are individual data points. WAB, Western Aphasia Battery.
FIGURE S4. The Relationships Between Network Damage Scores of the rest six ICs and AQ
(A-F) The scatter plots denote the relationship between patients’ AQ and the network damage scores of the IC #2, IC #3, IC #4, IC #5, IC #6, IC #7, respectively. The solid line represents the best linear fit with its 95% confidence interval (shaded area). Black dots are individual data points. AQ, Aphasia Quotient.
FIGURE S5. The Relationships Between Damaged Score Calculated by Classical Language Networks and AQ
(A-B) The scatter plots denote the relationship between patients’ AQ and the network damage scores of the language network defined in healthy people (A) and the language network defined by the large-sampled refined language network (B). The solid line represents the best linear fit with its 95% confidence interval (shaded area). Black dots are individual data points. AQ, Aphasia Quotient.

## Slide 11
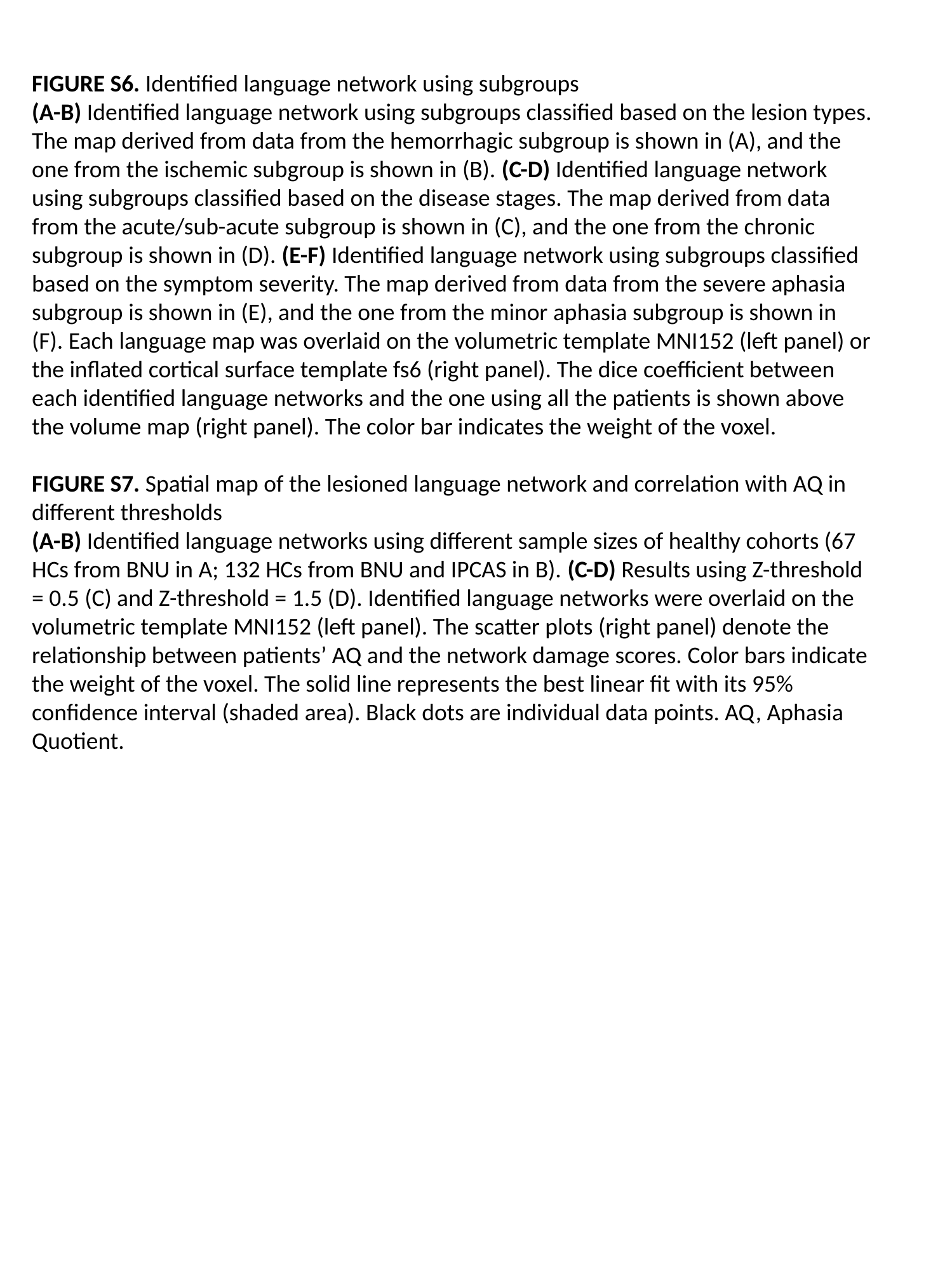

FIGURE S6. Identified language network using subgroups
(A-B) Identified language network using subgroups classified based on the lesion types. The map derived from data from the hemorrhagic subgroup is shown in (A), and the one from the ischemic subgroup is shown in (B). (C-D) Identified language network using subgroups classified based on the disease stages. The map derived from data from the acute/sub-acute subgroup is shown in (C), and the one from the chronic subgroup is shown in (D). (E-F) Identified language network using subgroups classified based on the symptom severity. The map derived from data from the severe aphasia subgroup is shown in (E), and the one from the minor aphasia subgroup is shown in (F). Each language map was overlaid on the volumetric template MNI152 (left panel) or the inflated cortical surface template fs6 (right panel). The dice coefficient between each identified language networks and the one using all the patients is shown above the volume map (right panel). The color bar indicates the weight of the voxel.
FIGURE S7. Spatial map of the lesioned language network and correlation with AQ in different thresholds
(A-B) Identified language networks using different sample sizes of healthy cohorts (67 HCs from BNU in A; 132 HCs from BNU and IPCAS in B). (C-D) Results using Z-threshold = 0.5 (C) and Z-threshold = 1.5 (D). Identified language networks were overlaid on the volumetric template MNI152 (left panel). The scatter plots (right panel) denote the relationship between patients’ AQ and the network damage scores. Color bars indicate the weight of the voxel. The solid line represents the best linear fit with its 95% confidence interval (shaded area). Black dots are individual data points. AQ, Aphasia Quotient.
